# ‘BRICS without straw’? A systematic literature review of newly emerging economies’ influence in global health

**DOI:** 10.1186/1744-8603-9-15

**Published:** 2013-04-15

**Authors:** Andrew Harmer, Yina Xiao, Eduardo Missoni, Fabrizio Tediosi

**Affiliations:** 1 Global Public Health Unit, Social Policy, School of Social & Political Science, University of Edinburgh, 15a George Square, Edinburgh, Scotland, EH8 9LD, UK; 2 Global Health and Development Area, Centre for Research on Health and Social Care Management (CERGAS), Bocconi University, via Roentgen 1, Milan, 20136, Italy; 3 Swiss Tropical and Public Health Institute, University of Basel, Basel, Switzerland

## Abstract

**Background:**

Since 2010, five newly emerging economies collectively known as ‘BRICS’ (Brazil, India, Russia, China and South Africa) have caught the imagination, and scholarly attention, of political scientists, economists and development specialists. The prospect of a unified geopolitical bloc, consciously seeking to re-frame international (and global) health development with a new set of ideas and values, has also, if belatedly, begun to attract the attention of the global health community. But what influence, if any, do the BRICS wield in global health, and, if they do wield influence, how has that influence been conceptualized and recorded in the literature?

**Methods:**

We conducted a systematic literature review in (March-December 2012) of documents retrieved from the databases EMBASE, PubMed/Medline, Global Health, and Google Scholar, and the websites of relevant international organisations, research institutions and philanthropic organisations. The results were synthesised using a framework of influence developed for the review from the political science literature.

**Results:**

Our initial search of databases and websites yielded 887 documents. Exclusion criteria narrowed the number of documents to 71 journal articles and 23 reports. Two researchers using an agreed set of inclusion criteria independently screened the 94 documents, leaving just 7 documents. We found just one document that provided sustained analysis of the BRICS’ collective influence; the overwhelming tendency was to describe individual BRICS countries influence. Although influence was predominantly framed by BRICS countries’ material capability, there were examples of institutional and ideational influence - particularly from Brazil. Individual BRICS countries were primarily ‘opportunity seekers’ and region mobilisers but with potential to become ‘issue leaders’ and region organisers.

**Conclusion:**

Though small in number, the written output on BRICS influence in global health has increased significantly since a similar review conducted in 2010 found just one study. Whilst it may still be ‘early days’ for newly-emerging economies influence in global health to have matured, we argue that there is scope to further develop the concept of influence in global health, but also to better understand the ontology of groups of countries such as BRICS. The BRICS have made a number of important commitments towards reforming global health, but if they are to be more than a memorable acronym they need to start putting those collective commitments into action. Keywords BRICS, global health, influence, newly emerging economies, Brazil, Russia, India, China, South Africa.

## Introduction

Following a decade of sustained growth in development assistance for health, new data suggests that global health financing has entered a period of ‘no-growth’, attributable in large part to the ongoing global economic crisis [1]. As Western economies stagnate, a number of ‘Eastern’ and ‘Southern’ economies continue to buck the trend, with one particular group of economies ‘the BRICS’ (Brazil, Russia, India, China and South Africa) reporting an average 6% growth between 2000 and 2010 [2]. Not surprisingly, therefore, international and global health policy makers are beginning to recognize these emerging economies as increasingly important actors. For example, Director of the World Health Organisation Margaret Chan has commented that the “… BRICS represents a block of countries with a … great potential to move global public health in the right direction … towards reducing the current vast gaps in health outcomes and introducing greater fairness in the way the benefits of medical and scientific progress are distributed …” [3]. In this paper we begin to unpack the ‘great potential’ of the BRICS, focusing specifically on their influence in global health.

In July 2011, Health Ministers of the Federative Republic of Brazil, the Russian Federation, the Republic of India, the People’s Republic of China and the Republic of South Africa, met in Beijing, China, for the First ‘BRICS’ Health Ministers’ Meeting^a^. Ministers committed, in what has subsequently become known as the ‘Beijing Declaration’, to initiate, champion and support a raft of global health measures [4]. Moreover, Health Ministers agreed to “promote BRICS as a forum of coordination, cooperation and consultation on relevant matters related to global public health” [4]. On the 22nd May 2012, BRICS’ Health Ministers met again in Geneva to discuss cooperation on health issues for their citizens “as well as the world at large” [5].

Attention has shifted to these five countries because, individually, they have made impressive – and increasing – contributions to international development. The prospect of a unified geopolitical bloc, consciously seeking to re-frame international (and global) health development with a new set of ideas and values, has begun to attract the attention of health researchers, policy makers and activists. The head of the Joint United Nations Programme on HIV/AIDS has lauded the BRICS countries for “bringing a new voice, a new perspective and new solutions to today’s global challenges” [6]. BRICS has also proven irresistible for political scientists, international relations scholars and economists seeking to understand the influence of this collection of countries in world affairs [7-9].

Despite this heightened interest in the BRICS and global health, to our knowledge, no study has sought to systematically approach the question of their influence. We asked: How is the influence of the BRICS *as a collective* in global health understood? As a first step towards answering that question, we conducted a systematic review of the literature.

## Methods

**Figure 1:**
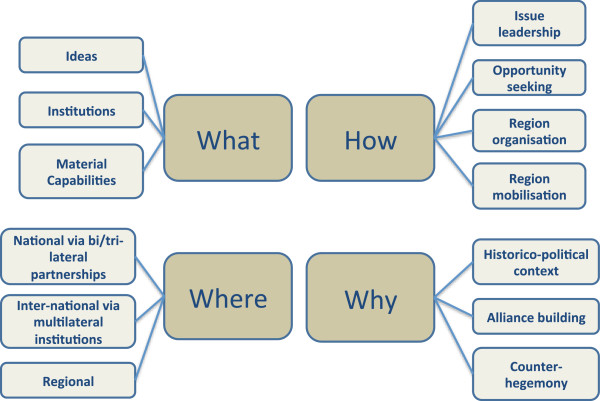
Anatomy of influence.

Our review was informed by two studies that usefully disaggregate the various components of influence [9,10]. We combined their analyses to produce one overarching framework that we used to structure our results (Figure 1). To summarise, our framework draws on a distinction familiar to International Relations scholars between ideas, institutions and material capabilities [10]. We also incorporated four ‘modes of international engagement’: issue leadership, opportunity seeking, region organising, and region mobilising [9]. Our framework generated four sub-questions: what kind of influence is exercised, how is it exercised, where is it exercised, and why? We did not, however, attempt to quantify how much influence the BRICS command.

**Figure 2:**
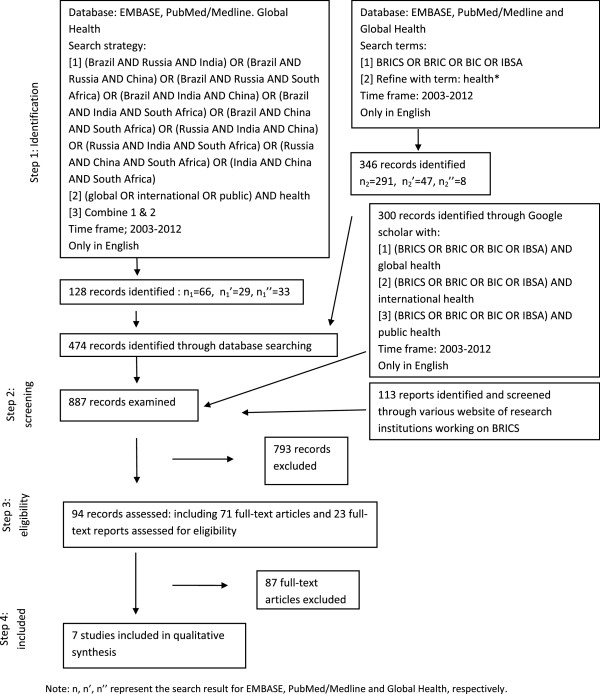
Flowchart.

A systematic literature review was conducted during March to December 2012. Our understanding of ‘global health’ was strongly informed by Bozorgmehr [11], where global health refers to: “supraterritorial links between the social determinants of health located at points anywhere on earth” [11], page 6. We began with a broad search of the databases EMBASE, Medline/PubMed, and Global Health. To ensure that we cast our net sufficiently wide, we used the key words ‘global health’, ‘international health’, and ‘public health’. Our research question required us to focus on the collective group ‘the BRICS’. As a first step, we designed a search string that combined the various combinations of at least three of the five BRICS countries (Figure 2). Whilst each of the BRICS has a long history in development cooperation, the significance of their collective influence arguably began with the birth of IBSA in 2003. Consequently, we limited our search to the period 2003–2012. We also limited our search to studies written in English.

We then conducted a separate search for articles using the keywords: BRICS, BRIC, BIC, IBSA, and refined the result with the term health*. All documents were imported into Endnote and duplications removed. As a further check, we conducted three searches using Google Scholar: [1]*global health + BRICS;*[2]*international health + BRICS;*[3]*public health + BRICS.* All searches were limited to articles (anywhere in the document) and to the time period 2003–12. Because Google Scholar retrieved an unmanageable number of hits, we limited our attention to the first 100 documents (sorted by relevance) from each of the three searches. The titles and abstracts were reviewed and exclusion criteria applied. Clinical trials, articles limited to domestic analysis, and studies that reported global epidemiology data (for example, global burden of disease reports) were excluded. Articles without a health focus were also excluded. Articles archived for further eligibility assessment.

Thirdly, we conducted a search of the websites of relevant international organisations (WHO, UNICEF, UNAIDS, UNPFA, IMF, OECD and the World Bank – please see Additional file 1 for a full list of acronyms), public-private partnerships (Global Fund to fight AIDS, Tuberculosis and Malaria, GAVI Alliance, UNITAID), national aid agencies (DFID, GIZ, USAID), independent research institutions (CGD, IHME, ODI, CSIS, IDRC, ORF, GHSi), and private philanthropy organisations (Bill & Melinda Gates foundation, Clinton Health Access Initiative). We excluded summary reports (those that did not have either an executive summary or a description of methodology) and reports of general global health issues that did not have a significant BRICS component. Reports containing single country analyses were also excluded.

Two researchers conducted an independent full-text review of the remaining journal articles and reports. Papers were only retained if they contained substantive qualitative and/or quantitative analysis or comparison of the BRICS as a collective bloc. Archives were compared and the final set of documents agreed. As a final check, the bibliographies of these documents were reviewed to ensure all relevant documents were included. No additional documents were added (Table 1).

**Table 1:** Key documents on BRICS economies and global health

**Author(s)/Editor(s)**	**Title**	**Focus countries**	**Document type**	**Published by**	**Geographical location of authors’ institution (country)**	**Year of publication**
Bliss, K (Ed)	Key Players in Global Health: How Brazil, Russia, India, China and South Africa are influencing the game [12].	B, R, I, C, S	Report	CSIS Global Health Policy Centre	USA	2010
Bliss, K	Health Diplomacy of Foreign Governments [13]	B, C, R	Report	CSIS Global Health Policy Centre	USA	2011
Tytel B and Callahan K (Eds)	Shifting Paradigm: How the BRICS are reshaping global health and development [14]	B, R, I, C, S	Report	Global Health Strategies Initiative (GHSi)	USA; Brazil, Russia, India, China, South Africa, UK	2012
Gomez, E	The Politics of Receptivity and Resistance: How Brazil, India, China, and Russia Strategically use the International health Community in Response to HIV/AIDS: A Theory [15]	B, R, I, C	Journal article	Global Health Governance 3:1	USA	2009
Kirton J, Larionova M and Alagh Y (Eds)	BRICS New Delhi Summit [16]	B, R, I, C, S	Summit Report	BRICS Research Group, Uni. Of Toronto	Canada	2012
Ruger, J and Noray, N	Emerging and transitioning countries’ role in global health [17]	B, R, I, C	Journal article	St. Louis University Journal of Health & Law 3:253	USA	2010
Yu, P	Access to Medicines, BRICS Alliances, and Collective Action [18]	B, R, I, C, S	Journal article	American Journal of Law and Medicine	USA, China	2008

## Results

Our literature review identified 94 documents satisfying broad inclusion criteria (71 journal articles and 23 reports) and just 7 documents satisfying the more precise criteria described above. Though few in number, it represents a significant increase in scholarly output: a literature review on this topic conducted for a study published in 2010 found just one study with the key words ‘BRIC’ and ‘public health’ [17]. The final 7 documents we identified provided the ‘dataset’ on which we based our analysis.

### What kind of influence do BRICS countries wield in global health?

#### Ideational influence

Separating ideational factors in the dataset from other elements of influence presented a challenge (Figure 1). After all, ideas underpin and inform priority setting, strategies, and mechanisms of influence. We therefore conducted a text search of the 7 documents for words that might point to an explicit ideational influence: ‘idea[s]’, ‘ideational’, ‘ideology’, ‘discourse’, ‘philosophy’, and ‘belief’.

Four studies analysed BRICS countries ideational influence on global health, with the bulk of the analysis reserved for just one country – Brazil [12-15]. The idea of health as a human right “permeates Brazil’s global health engagement” [13], page 6, as did the idea of “health in all policies” [12], page 13. The idea of rights was, according to one study, strong enough to eclipse economic drivers such as economic gain and trade considerations [12], page 3. South-South, triangular, and horizontal cooperation – all of which Brazil practiced and advocated – were also presented as ideational factors. Two studies discussed BRICS countries’ philosophy towards engagement with other countries [12,14]. Complementing previous analyses [12,13], the GHSi study described Brazil’s “core philosophy of availability and access” [14], page 25. The authors of the GHSi study also compared India and China’s philosophies towards international relations: India adopted a “demand-driven, horizontal philosophy” [14], page 47 while China was “guided by a philosophy of ‘mutually- beneficial’ development” with the aim of promoting self-sufficiency in recipient countries [14], page 9.

Conversely, ideational factors also motivated at least one of the BRICS countries to resists efforts by the international health community to influence its national health policies. For example, Russia resisted international recommendations to implement harm reduction strategies for AIDS treatment – a resistance that had its roots in an entrenched “conservative moral belief” [15], page 15.

#### Institutional influence

The term ‘institution’ describes national, international and multi-lateral organisations, but also the rules that structure the way societies interact (for example, rules of procurement or disease surveillance) [19]. Thus we can describe institutions as either organizational or rule-based. Six studies described the influence of BRICS countries’ institutions in global health [12-15,17,18]. Of these, five also described rule-based ‘social’ institutions [12-15,17].

In terms of organisational influence, various public, private and public-private organisations were identified in the literature (Table 2). At the national level, the Brazilian development agency Agencia Brasileria da Cooperação (ABC) was the most widely cited institution. Although 16% of ABC’s $30 million total budget in 2010 was directed towards health projects, “the lion’s share of international cooperation” in health was conducted by the Rio-based public-private partnership Fundação Oswaldo Cruz (Fiocruz) [12], pages 4–5. Historically, Brazil supported multilateral institutions, such as the World Health Organisation (WHO). Whilst continuing to support those institutions, in recent years the country had sought to promote “alternative institutions” such as the G20 rather than traditional institutions that it viewed as dominated by the global North, with the aim of “undermining the traditional international political status quo” [12], page 13.

**Table 2:** Selected BRICS countries’ institutions

**Country**	**Name of institution**	**Public or private**	**Sphere of influence**	**Selected health issues**
Brazil	Agência Brasileira do Cooperação (ABC)	Public	Regional bilateral; Africa	Malaria; HIV/AIDS; universal health care; nutrition; human milk banks; surveillance; technology transfer
	Oswaldo Cruz Foundation (Fiocruz)	Public	Latin America	R&D; production of vaccines, reagents, drugs and diagnostics; human resource training; nutrition
Russia	Roscooperation (inter-ministerial commission)	Public	Russia’s fledging international aid agency	International health development
	Medical schools	Public and academic sector	CIS and Asia	Training for 20,000 medical students annually; disease surveillance
India	Public health foundation of India	Public-private funded	Nationally and bilaterally with US and UK	Established a network of public health schools
	Aravind Eye Care	Private	Nationally; China and Egypt	Technical assistance for eye care
	Dept. of Biotechnology	Public	National	Biotechnology Innovation
	National AIDS Research Institute	Public	National	Medical research
China	China National Biotec Group (CNBG)	Public-private	National	Vaccine innovation
	Shanghai Dahua Pharmaceutical Co.	Public-private	National	Women’s condom
South Africa	Treatment Action Campaign; AIDS Law Project	Not-for-profit non-government organization	National	Activism for access to drugs
	Desmond Tutu AIDS Centre	Public	National and bilateral with US and Europe	R&D and clinical research focused on infectious diseases

In terms of both organisational and rule-based institutions, Russia’s “institutional architecture” was “very much a work in progress”, although a national development agency – RUSAID – was expected in 2012 [12], page 36. In contrast to the other BRICS countries, the Russian government was reliant on multilateral institutions such as the World Bank and United Nations Development Program (UNDP) to manage the transfer of its overseas development assistance. For certain health issues, such as infectious disease surveillance, Russia was able to take advantage of a strong institutional legacy inherited from the days of the Soviet Union. In other respects, notably the absence of an effective regulatory, legal, and institutional framework for aid delivery, Russia was “in search of its destiny” – wanting to use health as a foreign policy tool but lacking the institutional capacity to achieve its ambitions [12], page 40.

India had a strong ‘government-dominated’ institutional profile, but no single ministry representing its health concerns at international fora. Consequently, its health representatives were generally not regarded as “strategic leaders” – unlike the country’s economic representatives, who commanded more attention globally [12], page 26. In July 2012, India announced that it was establishing a $15 bn international foreign aid agency [20]. Nevertheless, India’s institutional ‘energy’ remained less in “conventional institutional frameworks” such as the World Bank or the World Health Organisation, and more in “unique institutions” such as the Public Health Foundation of India or private institutions such as Aravind Eye Care – described as “the most rapidly expanding area of India’s international engagement on health” [12], page 28.

As of July 2012, China had no specialised agency to manage its official development assistance. One study maintained that China’s ambitions to extend its global health outreach were being thwarted by the country’s limited bureaucratic capacity to manage overseas work, and that could “place limits on the extent to which China will emerge as a global health leader” [13], page 4. Social institutions were also influential at different periods in China’s history. Thus in the 1990s, China’s Premier was free from legal and bureaucrat constraints, and chose not to engage the international community [9]. In the years following, Chinese leaders have become less autonomous from their country’s institutions, and successive governments have returned to their tradition of international cooperation, particularly in disease surveillance [15].

As with India and Russia, South Africa had only just announced the launch of a development aid agency at the time of writing. Of all the BRICS countries, South Africa was one of just two BRICS countries (with Brazil) whose non-governmental organisations (NGO) were cited as being influential. South Africa’s Treatment Action Campaign, for example, has successfully campaigned to reduce the price of medicines, prevented hundreds of thousands of HIV- related deaths, but also forced significant additional resources into South Africa’s health system and towards the country’s poor [21].

#### Material influence

Each of the seven studies commented on the material influence of the five BRICS countries (Table 3). Four studies in the dataset observed that domestic economic and political context were the drivers behind BRICS countries’ international and global outreach: “the discussions revealed that domestic health and political conditions in each country exert a profound influence on how each one’s global health outreach is structured and publicised” [13], page 2. While economic power was often cited as a source of BRICS countries’ influence, one study cautioned against overstating its importance: “the effectiveness of the BRICS coalition is not dependent on the future economic strength of its members” [18], page 358. A combination of raw materials, technical capacity, manufacturing conditions, and new markets, could be sufficient, the author argues, for the BRICS to re-align Western-driven global Intellectual Property regimes towards greater access to essential medicines for developing countries [18], *ibid*. For example, the BRICS and less-developed countries could take advantage of their capacity to provide generic versions of on-patent drugs and ability to supply active pharmaceutical ingredients to domestic manufacturers to “threaten the survival of major pharmaceutical manufacturers in the developed world” [18], *ibid*. Were such a “face-off” to ever to materialise, then the impact of the BRICS coalition on the access-to-medicines debate would likely be “considerable” [18], *ibid*.

**Table 3:** Selected data on BRICS countries material contribution to international health

	**Brazil**	**Russia**	**India**	**China**	**South Africa**
Year foreign assistance programs started [14]	1960	1955	1964	1950	1968
Foreign Aid Focus [14]	Health; Education; Agriculture	Health; Education; Food Security	Infrastructure; Information technology; Training and Capacity building	Infrastructure; Industrial development; Energy resources development	Peacekeeping; Democracy promotion
Foreign ‘aid’ $US million (last year available) [22]	362 (2009)	472.3 (2010)	639.1 (2010)	2010.6 (2010)	98.4 (2010)
Health component as % of foreign ‘aid’ (last year/period available)*	16.6% (not specified at source) [23]	25% (2006–10) [8]	Not available	Not available**	Not available***
Preferred channel for health ‘aid’ [14]	Trilateral	Multilateral	Primarily bilateral	Primarily bilateral	Bilateral

* Each of the BRICS countries has its own preferred lexicon to describe its international ‘aid’ (e.g. Brazil – ‘international cooperation’; Russia – ‘foreign assistance’; India – ‘demand-driven’).

** Estimated health assistance to Africa 2007–11 was $US 757 m [14], page 63.

*** The Mbeki administration gave US$10 million to the Global Fund between 2003–2007 and in 2006 pledged US$20 million to the GAVI Alliance for a 20 year period [14] page 75.

### How do BRICS countries influence global health?

In their study of BRICS and the global politics of development, Hau et. al. provide four modes of international engagement: issue leadership; opportunity seeking; region organising and region mobilising [9]. Which, if any, of these four modes applies to BRICS countries seeking to influence the international and/or global health agenda?

#### Issue leaders; opportunity seekers

According to Hau et. al., issue leaders and opportunity seekers operate at the global level. Issue leaders are multilateralist to the extent that they engage in policymaking in international organisations [9]. They provide intellectual leadership, technical support and political convening facilities, and thus derive their influence from the use of coalitions and consensus building [9], page 190. Opportunity seekers differ from issue leaders in that they seek opportunities to establish bilateral relations with other countries rather than multilateral relations [9], page 191.

BRICS countries’ multilateral engagement in global health was discussed in four studies, though limited almost exclusively to economic engagement [12-14,18]. There were a number of examples from the dataset of BRICS political multilateral engagement, though little substantive analysis. For example, India was described as “an outspoken member” of the G20 [14], page 44, although its health representatives were not yet regarded as “strategic leaders”, while Russia was “active” in both the G8 and G20 [13], page 15. Brazil appeared to enjoy a schizophrenic approach to multilateralism: on the one hand it demonstrated strong leadership in securing the Framework Convention on Tobacco Control, but was also keen to nurture relations of “solidarity” with countries of the ‘global South’ with “hopes of undermining” traditional political structures dominated by the global north [13], page 15. China’s geopolitical concerns, notably territorial disputes with Taiwan, constrained its ability to maneuver within multilateral organisations [18], page 22.

#### Region organising; region mobilising

Hau et. al.’s framework also provides two strategies that BRICS countries might adopt in order to influence regional policy: one is by leading the organization of regional fora for health; another is by mobilising strategic and economic ties with neighbouring countries through bilateral and/or multilateral agreements [9], page 192.

Four studies reported on BRICS countries’ regional influence [12-15]. Examples of regional organising included China using its regional dominance of ASEAN to set up a China-ASEAN Fund for Public Health to support technical training for professionals from ASEAN member states [13], page 4; Brazil fostering cooperative efforts with countries within the Community of Portuguese-Speaking Countries, as well as the Union of South American Countries to strengthen health education, technical assistance, and research [13], page 7; Brazil working with its regional partners to improve disease surveillance capability – what one study described as an “epidemiological shield” [13], page 6; and Russia’s involvement in health-related discussions through the Shanghai Cooperation Organization and the Health Working Group of the Asia-Pacific Economic Cooperation [13], page 5. Each of the BRICS countries was engaged in regional disease surveillance networks, although were not necessarily leading those networks [14], page 91.

Both India and China began their international health cooperation as region mobilisers but since the middle of the last century have sought to expand their global outreach into Africa and Asia, and thereby behaving more like opportunity seekers [24], page 574. One study noted that China and Russia signed a 2001 Treaty of Good-Neighborliness and Friendly Cooperation in 2001, using that formal agreement to promote humanitarian cooperation in areas that included health care [12], page 18. China was also reported as “actively promoting” health cooperation with its Association of South-East Asian Nations neighbours. Prior to 2003 only Nepal received financial assistance from India. Since 2003 India’s priority has been to strengthen neighbourly relations with Afghanistan, Nepal, Bangladesh and Sri-Lanka: global outreach into Africa was of secondary importance [12], page 27. In this respect, India was behaving more as a region mobiliser than opportunity seeker. Brazil, too, has strengthened bilateral relations with its neighbours to improve access to health care services, facilitate joint action on epidemiology, health and environmental surveillance, and to eliminate specific diseases such as onchocersiasis [12], page 11.

### Where do BRICS countries seek influence?

All of the studies discussed individual BRICS countries’ international cooperation for health, although most of the commentary was limited to four Reports [12-14,18]. Bilaterally, Lusophone countries were the traditional recipients of Brazilian health assistance, with Mozambique, Timor-Leste and Guinea Bissau topping the list of beneficiaries between 2005 and 2010. Countries in Latin America and the Caribbean, particularly Haiti, Paraguay and Guatemala were also important recipients. Russia’s limited bilateral assistance was almost exclusively focused on the Commonwealth of Independent States, particularly neighbouring countries – reflecting Russia’s concern for the cross-border spread of diseases. India’s official aid programs were primarily focused on bilateral cooperation with its neighbours Bhutan, Nepal and Afghanistan. Although health was not a priority for its foreign aid agenda, a total of $100 million aid had been committed to South Asia, Southeast Asia and Africa since 2009 [14].

China’s aid to Africa, Latin America and Southeast Asia increased from less than $1 billion in 2002 to an estimated $25 billion in 2007 [14]. In 2009, Chinese medical team totalled 21,000 in 69 countries. Most of China’s global health ‘outreach’ is directed towards Africa. Between 2010–2012, China provided medical equipment and drugs to 30 hospitals, and Chinese funding was used to build 30 prevention and treatment centres for malaria, serving approximately 73 million people [25]. Health infrastructure building and human resource training were also provided [23,26].

South Africa’s bilateral health aid went almost entirely to other African nations in the form of grants or technical support, mostly focused on the member states of the South African Development Community. However, the country’s Department for International Development and Cooperation (DIRCO) has stated its intent to continue supporting integral development assistance programmes such as the India-Brazil-South Africa (IBSA) Fund for Poverty and Hunger Alleviation, the African Development Bank and the Southern African Customs Union [23]. As with other BRICS countries, South Africa’s primary concern is to meet its own domestic health needs [12,14].

### Why do BRICS countries seek influence?

One study identified “several common threads” that explained BRICS countries global health outreach: “each country’s engagement is driven by its history and political outlook; framed by its view of how global health advances its own sovereign interests; and shaped by the image it seeks to project regionally and internationally” [12], page 15. We return to these common threads in the discussion. The idea of “symbolic capital” was also raised in another study [13], page 2. Again, we return to this motivation below. More familiar motivations – to ensure alignment with national health priorities; economic advantage; regional stability; reputation-building; altruism; and as a foreign policy tool – were also cited in various studies [13,14]. The importance of social equity was noted in a number of studies, but this was a concept more closely aligned to the values of the IBSA countries [12], and Brazil in particular [14].

One point to note is that whilst these studies cited various reasons why BRICS countries sought influence, very few developed those assertions into cogent analysis. For example, a number of studies asserted that BRICS countries could strengthen their influence through alliances [12]. Only one study developed those assertions into a sustained argument [18]. The author of that study argued that a complete or even partial BRICS alliance could prove an effective “countervailing force” against European Community and United States efforts “to ratchet up global intellectual property standards”, with beneficial consequences for developing countries’ access to medicines [18], page 373.

Another motivation for seeking influence expressed in a number of the studies was that BRICS countries were deliberately cultivating and promoting a new model of international cooperation. BRICS countries were described as presenting a novel perspective on international relations that challenged Western-centric approaches to development assistance for health [18]. Chief amongst the elements of that new world order were South-South cooperation, equity, trilateralism, and adoption of a new development lexicon that emphasised partnership, and mutuality. However, it should be emphasised that not all of the BRICS countries supported all of those elements, and not necessarily in equal measure.

Several studies discussed BRICS countries’ commitment to South-South cooperation. Brazil, India, China and South Africa were enthusiastic supporters [14], though not Russia – which preferred to engage in North-North and North–south relations. China was particularly enthusiastic in its support for South-South cooperation, regarding it as “a way of compensating for the wrong-doings of the North in the South” [24], page 580. Although trilateralism was identified in a number of studies, there were inconsistencies in reporting [14], page 23. Commitment to partnership and mutuality to BRICS countries was also frequently cited, an observation echoed by non-health studies of BRICS. Kragelund, for example, has argued that the “re-emergence” of such principles “challenges traditional donors’ authority to set the standards and norms of development aid in the future” [27], page 599.

## Discussion and avenues for further research

Given the consistent attention afforded the health ministerial activity of the BRICS in the grey literature as well as extensive coverage in the media, one might have expected to find more than the 7 studies we identified that satisfied our inclusion criteria. Of these, only 3 were peer-reviewed journal articles. What might explain this paucity of literature? One study concluded that it was still ‘early days’ for the BRICS and global health: “The BRICS have declared health collaboration a priority, but they have not yet begun to work collectively to enhance the impact of their assistance programs” [14], page 11. Oliver Stuenkel has made a similar point:

*“Very little serious academic writing has been published so far on the BRICS, simply because it is such a recent phenomenon and many academics are reluctant to speculate and prefer to wait for tangible evidence before putting their thoughts on paper”*[28].

The BRICS have only been a collective group since 2010, so it is perhaps unrealistic to expect any coordinated output beyond the level of a communiqué. The BRIC countries have been together for longer, but health has simply not been a sector priority – at least not until 2011 and the 1st Health Ministers Meeting. Indeed, it has taken this bloc of countries 7 years to get to the point where a new financing mechanism – the BRICS bank – is being publicly mooted as a serious possibility. So we should not be surprised that a ‘softer’ issue such as health is being negotiated at a more measured pace.

An important, and unexpected, finding from our review was that the acronym BRICS was described using both the indefinite and definite articles, which were often used interchangeably within the same document. Thus Brazil was *a* BRICS country but also a member of *the* BRICS. This raised an interesting ontological question: what was the unit of analysis – what was BRICS?

Admittedly, we are not the first to ask this question. In the field of International Relations, for example, Armijo asks whether the term ‘BRICs countries’ is “a viable analytical category” [7]. The author notes, as we do, that the four BRICs (Armijo does not include South Africa in her analysis) have quite different domestic political institutions, international goals, and economic structures. She argues that the BRICs could be considered “an analytically viable set… [if]…some other economic characteristic plausibly distinguished the four from the larger set of developing and post-communist countries known as emerging market economies” [7], page 39. Armijo found no such distinguishing characteristic and thus concluded that the BRICs was “strictly speaking, a mirage—but one that nonetheless has provided considerable insight” [7], page 40. Our literature review concurs with this general conclusion.

Each of the studies we identified drew attention to the political, economic and ideational differences between the individual countries, with some analysis of common themes [12]. As noted above, just one study provided *sustained* analysis of the BRICS’ collective influence [18]. The other studies implicitly assumed that the BRICS did, or would, influence global health as a bloc in the future, without considering critically what influence might mean.

One of the studies we identified made the point that while government institutions were important actors both nationally and internationally, institutional influence was “not just about governments” [12], page 32*.* However, notable by its absence are analyses of BRICS’ non-state actors – particularly civil society, but also the private sector. International development scholars and geographers have noted the extent to which BRICS countries encouraged and supported the reach of these sectors beyond the domestic sphere [29]. Further research is required on the role of emerging economies’ non-state actors in supporting global health priorities such as universal health care.

Our findings suggest that we have arrived at an intersection between multiple disciplines: international development, international relations, and global health. Of the three journal articles in our dataset, just one article was from a public health journal: the remaining two were published in development and law journals. BRICS and global health is a subject amenable to inter-disciplinary analysis: analysis of influence, for example, is a mainstay of both international development and international relations [9,10,30,31] and global health could draw on this body of work. Conversely, analysis of the BRICS’ contribution to global health – or failure to contribute – could inform international relations and development understanding of the ontology, and identity, of BRICS.

Critical analysis of BRICS by the global health community is essential. It is not sufficient to assume that the BRICS will contribute to global health; an understanding their influence in global health would benefit from critical attention, drawing on insights developed within the major paradigms of International Relations – Realism, International Institutionalism, and Critical Theory [9,30,31]. In one of the seven studies we identified, the authors argued that, historical and political contexts aside, the five countries “frequently do collaborate as BRICs, IBSA, or BASIC on issues related to health” [12], page 6. However, we know very little about the nature of that collaboration or, more importantly, whether it was/is effective. We also found an uncritical presentation of the various elements of the ‘new model’ of the BRICS’ development assistance. The temptation to aggregate these various elements and present them as the BRICS approach is tempting but upon closer scrutiny it is clear that few of those elements are common to all five countries [29].

Furthermore, the consequences for global health of adopting ‘novel’ approaches to international relations such as South-South, or mutuality, or ‘demand-driven’ are not critically assessed in any of the studies. For example, many of the studies reported that BRICS countries’ global health agendas were driven by domestic priorities and/or regional concerns. Whilst that is understandable, how realistic is it to expect the BRICS to reflect more than a parochial interpretation of global health?

Values, such as Brazil and South Africa’s promotion of equity in global health, also warrant further attention. There are good reasons to wish for an equity-driven model. However, as the process of drafting the text of the key document of the 2012 Rio + 20 UN Conference on Sustainable Development (UNCSD) shows, inclusion of the word ‘equity’ was a source of diplomatic tension between emergent economies such as Brazil and Western economies such as the United States [32]. Other values such as transparency are a central tenet of Western conceptions of global governance, though China has shown reluctance to include it as a standard for South-South cooperation. As reported above, one study describes the BRICS alliance as presenting a ‘countervailing force’ to Western-dominated prescriptions for global health. On the one hand, it is possible that BRICS “may be able to establish, shape, and enlarge a pro-development negotiating agenda” and “help enlarge the policy space less developed countries need” [18], page 258. On the other, not aligning with those prescriptions may create a two-track development agenda that impedes diplomatic efforts to agree a needs-driven, rights-based, or even equitable global health agenda.

## Conclusion

In thinking about the future trajectory of BRICS’ influence in global health, we offer two contradictory interpretations of a well-known aphorism – ‘bricks without straw’^b^. On the one hand, successfully making bric[k]s without straw is a commendable (because difficult) achievement. An optimistic reading of the literature would point to the successful convening of four BRICS Summits and two BRICS Health Ministers Meetings as a positive first step that will, hopefully, result in concrete collective action for global health at some unspecified point in the future. With China now represented at the head of the World Health Organisation’s Executive Board, and both India and Brazil now making robust interventions at the World Health Assembly, one might conclude that BRICS have the potential to reconfigure Western-centric models of global health governance and development assistance.

On the other hand, a pessimistic reading of the aphorism might note that the absence of ‘straw’ is liable to render foundations unstable – much like building a house on sand. On this reading, BRICS are either incapable of cooperating or coordinating their actions or, worse, they may impede ongoing efforts by developed countries, jeopardising internationally agreed principles and norms.

Scholarly analysis of *the* BRICS (rather than individual BRICS countries) influence in global health has increased in the last five years, but remains very weakly understood. Explaining this deficit by arguing that it is ‘still early days’ or that there is little for researchers to investigate is not convincing. Our review identifies a wide range of issues that warrant further academic attention, not least further conceptual analysis of the nature and possibilities for influence in global health; the extent to which the BRICS are beginning to influence single global health issues (for example, reform of the World Health Organisation or Universal Health Coverage); and the role of the BRICS’ non-state actors (for example, the ‘new’ foundations). A sensible strategy would be to approach the BRICS, and other groupings of newly emerging economies (for example, ‘MIST’ – Mexico, Indonesia, South Korea, and Turkey) through an inter-disciplinary lens (development geographers, for example, are taking this analysis forward, and the health community might seek productive collaboration there).

Our review found little evidence to support the assertion that *the* BRICS are influencing global health, although individual BRICS countries are becoming more vocal and active in shaping, and indeed leading, global health movements such as Universal Health Coverage or generic drug production. The various Summits and meetings of BRICS health Ministers suggests that there *is* political will for collective action, with political leaders now recognising that there is an opportunity for their health ministers to move the agenda for global health in a new direction. The challenge is to build on that momentum and convert political will into action.

## Endnotes

^a^ Brazil, Russia, India and China first met as a geopolitical bloc in 2006, although the acronym ‘BRIC’ was first coined in 2001 by an economist from the investment bank Goldman Sachs. These four countries held their first Summit in 2009, repeated annually since, with the most recent held in New Delhi in March 2012. BRIC became BRICS in 2010 when South Africa was invited to join.

^b^ The proverb ‘you can’t make bricks without straw’ derives from an Old Testament story: when asked by Moses to free his slaves from their servitude, a slave-owning Pharaoh refused. He then made his slaves’ primary task – making bricks – more difficult by refusing them straw (a primary ingredient). Undaunted by this additional hardship, the Pharaoh’s slaves continued to produce well-made bricks.

## Competing interests

The authors declare that they have no competing interests.

## Authors’ contributions

AH and FT conceived the original idea for the review. AH designed the conceptual framework and wrote the final draft. AH and YX designed the literature review and conducted analysis of the results. YX, EM and FT provided comments on various drafts and contributed to writing the final manuscript. All authors have read and approved the final manuscript.

## Supplementary Material

Additional file 1: List of acronyms. Click here for file
