# Supplementary material for: ‘BRICS without straw’? A systematic literature review of newly emerging economies’ influence in global health
Source: Global Health. 2013 Apr 15;9:15. doi: 10.1186/1744-8603-9-15 (PMC3637153; doi:10.1186/1744-8603-9-15)
Supplement: Additional file 1: — List of acronyms. [file 1744-8603-9-15-S1.docx]

**Additional file 1: List of acronyms**

ABC: Agencia Brasileria da Cooperação (Brazilian development agency)

ASEAN: Association of Southeast Asian Nations

BASIC: Brazil, South Africa, India and China

BIC: Brazil, India and China

BRIC: Brazil, Russia, India and China

BRICS: Brazil, Russia, India, China and South Africa

CGD: Center for Global Development

CSIS: Center for Strategic and International Studies

DFID: The Department for International Development

DIRCO: Department for International Development and Cooperation

G20: Group of 20 (Argentina, Australia, Brazil, Canada, China, France, Germany, India, Indonesia, Italy, Japan, México, Russia, Saudi Arabia, South Africa, Korea, Turkey, the United Kingdom, United States and European Union)

G8: Group of 8 (United Kingdom, France, Russia, Germany, Japan, Italy, Canada, and the United States)

GHSi: Global Health Security Initiative

GIZ: Deutsche Gesellschaft für Internationale Zusammenarbeit, GmbH (German Society for International Cooperation)

IBSA: India, Brazil and South Africa

IDRC: International Development Research Centre

IHME: Institute for Health Metrics and Evalluation

IMF: International Monetary Fund

ODI: Overseas Development Institute

OECD: Organization for Economic Co-operation and Development

ORF: Observer Research Foundation

RUSAID: Russian Agency for International Development

UNAIDS: The Joint United Nations Programme on HIV and AIDSun

UNCSD: United Nations Conference on Sustainable Development

UNDP: The United Nations Development Programme

UNICEF: The United Nations Children’s Fund

UNPFA: The United Nations Population Fund

USAID: U.S. Agency for International Development

WHO: World Health Organization
